# Immersive Virtual Reality for Patient Education in Orthodontics: Cross-Sectional Feasibility and Acceptability Study

**DOI:** 10.2196/102686

**Published:** 2026-08-27

**Authors:** Yoo Jin Kim, Glenn Jou, Hyun Cho, Mathias Stephen Fallis, Glenn Sameshima

**Affiliations:** 1 Center for Advanced Dental Education, Department of Orthodontics, Saint Louis University, St. Louis, MO, United States; 2 Department of Pediatric Dentistry and Orthodontics, Herman Ostrow School of Dentistry of USC, University of Southern California, 925 W 34th St, Suite 312E, Los Angeles, CA, 90089-0641, United States, 1 213-740-4352; 3 Department of Media Arts and Technology, University of California, Santa Barbara, Santa Barbara, CA, United States

**Keywords:** virtual reality, extended reality, immersive technology, orthodontics, patient education, patient perception, serious games, digital health, feasibility study

## Abstract

**Background:**

Patient education is central to orthodontic care; however, most patients still learn about their malocclusion from conventional records, such as 2D image records. Immersive virtual reality (VR) allows patients to view and manipulate 3D dental models in a head-tracked environment, as part of the serious-games and extended reality family for health education. Evidence on patient-facing immersive VR in orthodontics remains limited, as most dental VR work has addressed student training rather than patient education.

**Objective:**

This feasibility study aimed to evaluate acceptance, perceived educational value, self-rated experience, ease of use, and self-reported preference regarding an immersive VR consultation room among patients undergoing orthodontic treatment.

**Methods:**

This cross-sectional, single-arm feasibility and acceptability study was conducted in a university orthodontic clinic between August and September 2024. Patients undergoing active orthodontic treatment completed a single, approximately 15-minute immersive VR educational experience using a Meta Quest 3 headset and a Unity3D-developed consultation room presenting a 3D rendering of each participant’s own intraoral scan, followed by an investigator-developed postexperience questionnaire. The questionnaire combined a Likert scale and two 10-point numeric items with open-ended prompts. Closed-ended responses were summarized as counts, percentages, and 95% CIs, and numeric items were summarized as means with 95% CIs; open-ended comments were grouped into themes. No inferential testing was performed.

**Results:**

Twenty-eight patients completed the study (mean age 21.6, SD 4.3, range 13‐33 y; 18 males, 10 females; 5 minors). Sixteen (57%) patients had no prior VR experience, 22 (79%) were interested in using VR to learn about their teeth, and 24 (86%) agreed that VR could help patients understand their teeth and braces. The mean overall experience rating was 7.9 (SD 1.6; 95% CI 7.3‐8.5) on a 10-point scale, and the mean ease-of-use rating was 6.6 (SD 2.6; 95% CI 5.7‐7.6). Twenty (71%; 95% Wilson score CI 53‐85) patients felt VR was a better learning modality than conventional 2D images; 19 (68%; 95% Wilson score CI 49‐82) preferred VR with the current setup, and 25 (89%; 95% Wilson score CI 73‐96) preferred VR under a hypothetically improved technology scenario. Positive comments emphasized interactivity, 3D visualization, and engagement; negative comments centered on controller difficulty, latency, and motion discomfort. Two (7%) patients reported mild, transient, motion-related discomfort; none discontinued.

**Conclusions:**

Patients were generally receptive to an immersive VR consultation room. This work is novel in evaluating patient-facing immersive VR using each patient’s own intraoral scan and in identifying actionable usability and motion-comfort barriers. Because the design was single arm and the comparison with conventional 2D image records was self-reported, the findings represent preliminary acceptability evidence, not learning effectiveness. If confirmed in more rigorous studies, immersive VR may serve as an adjunct to communication with patients undergoing orthodontic treatment, warranting controlled studies with an interactive flat-screen 3D comparator; validated usability, presence, and cybersickness instruments; and objective knowledge-gain measures.

## Introduction

### Background

Effective patient education is fundamental to orthodontic care because patients must understand their malocclusion, treatment goals, and expected outcomes to participate meaningfully in decision-making and long-term treatment adherence. Prior work in orthodontics has shown that patient satisfaction is shaped in large part by communication and by the extent to which patients feel involved in their care [1]. Digital tools that make treatment explanations clearer and more engaging may therefore improve the educational experience.

As orthodontics becomes increasingly digital, patients are also becoming more accustomed to technology-based visualization. Intraoral scanning and digital models, for example, have been shown to be acceptable to patients undergoing orthodontic treatment, and in some settings, preferred over conventional methods [2]. At the same time, the broader dental education literature suggests that virtual reality (VR) can improve engagement, confidence, and perceived educational value, although most published work has focused on student training rather than patient education [3-5]. Beyond dentistry, immersive VR has been found to be acceptable and feasible for education and engagement across diverse clinical populations, including users with limited prior VR experience [6,7]. Acceptability is nonetheless context- and population-specific, and evidence generated in other settings cannot be assumed to transfer to patients undergoing orthodontic treatment, who differ in typical age range, clinical context, and educational goals. Establishing acceptability in this population is therefore a necessary first step before larger controlled studies of educational effectiveness are warranted.

### Prior Work

Immersive VR offers a different way for patients to interact with dental information by allowing them to view and manipulate 3D models in a head-tracked virtual environment using handheld controllers [8]. Unlike conventional 2D images or static physical demonstrations, immersive VR may provide a more interactive and spatially intuitive way to understand tooth position, occlusion, and treatment concepts [9,10]. Immersive experiences of this kind, which combine 3D visualization with goal-directed interaction and feedback, sit within the broader family of serious games and extended reality (XR) tools designed for health education and behavior change rather than entertainment. In the present study, these defining features (guided, goal-directed model manipulation with audiovisual feedback rather than free play) serve as the basis for situating the experience within the literature.

Evidence specifically addressing immersive VR as a patient-facing educational modality in dentistry remains limited. A pilot study in cleft care (N=20) suggested that VR-based education may improve caregiver understanding and treatment acceptance [11]. More recent patient-facing dental literature has also suggested potential benefits of VR for oral health instruction and for improving the patient experience in anxious dental populations, including a scoping review of VR for dental anxiety [12] and a randomized trial of VR-based toothbrushing instruction in adults (N=30) [13]. However, these applications remain distinct from patient education in orthodontics, and a recent scoping review in orthodontics identified patient engagement as a promising but still understudied use of XR technologies [14]. Recent orthodontic XR applications have largely targeted student and trainee education, such as VR for undergraduate orthodontic teaching [15] and web-based augmented-reality tools for orthodontic biomechanics [16], rather than communication with patients themselves. To our knowledge, patient-facing immersive VR that presents a patient’s own intraoral scan for orthodontic education has not previously been evaluated for acceptability.

### Study Objectives

Against this background, the aim of this feasibility study was to evaluate acceptance of an immersive VR consultation room among patients undergoing orthodontic treatment and to describe their perceptions of usability, educational value, and preference compared to conventional 2D image records. Findings are intended to inform iterative refinement of the system and the design of future controlled studies measuring objective knowledge gain.

## Methods

### Study Design and Setting

This was a cross-sectional, single-arm feasibility survey conducted in the orthodontic clinic at the Herman Ostrow School of Dentistry of the University of Southern California (USC) between August and September 2024. Current patients undergoing active orthodontic treatment were recruited during routine clinic visits and invited to complete a one-time immersive VR educational experience followed by a postexperience survey. The full questionnaire is available in Multimedia Appendix 1. This early-phase feasibility and acceptability evaluation is reported in accordance with the RATE-XR (Reporting for the early-phase clinical evaluation of applications using extended reality) reporting guideline for XR applications [17].

### Inclusion and Exclusion Criteria

Inclusion criteria were (1) patients currently undergoing active orthodontic treatment in the USC orthodontic clinic, (2) recruited during a routine clinic visit within the study period, (3) able to understand spoken English instructions for the VR experience and the survey, and (4) able and willing to provide informed consent, or, for minors, assent, together with parental or guardian permission. Exclusion criteria were (1) inability to wear a head-mounted VR display, (2) a self-reported history of severe motion sickness or a vestibular disorder, and (3) any acute medical condition precluding safe participation, as judged by the supervising clinician. No additional exclusion criteria were applied. Participation was voluntary, and declining the activity had no effect on routine clinical care.

### Participant Characteristics

A total of 28 patients completed the study. The sample had a mean age of 21.6 (SD 4.3, range 13‐33) years and included 18 male and 10 female participants. Five participants were minors (<18 y). All participants had previously reviewed their malocclusions using conventional 2D images of models of their teeth as part of routine orthodontic care. These conventional records were noninteractive and could not be manipulated in 3D by the patient.

### Sampling Procedures

Participants were recruited using consecutive convenience sampling. Eligible patients undergoing orthodontic treatment attending routine visits at the USC Orthodontic Clinic during the study period (between August and September 2024) were approached in the order in which they presented and invited to participate until recruitment concluded; therefore, self-selection was possible. The numbers of patients approached and those who declined participation were not recorded prospectively; therefore, the percentage of those approached who participated is unknown. No advertising or external recruitment was used; participation was voluntary; and no compensation was provided. Institutional review board approval and consent procedures are described in the *Ethical Considerations* section.

### Sample Size, Power, and Precision

As an early feasibility and acceptability study, no formal sample-size calculation was performed and no hypothesis testing was planned. The achieved sample of 28 participants is consistent with the modest sample sizes of comparable feasibility and pilot studies, whose purpose is to assess acceptability and to inform effect-size and sample-size assumptions for a future controlled trial rather than to support inferential conclusions. CIs are reported throughout to convey the limited precision of estimates derived from this sample.

### Immersive VR Intervention

**Figure 1.**
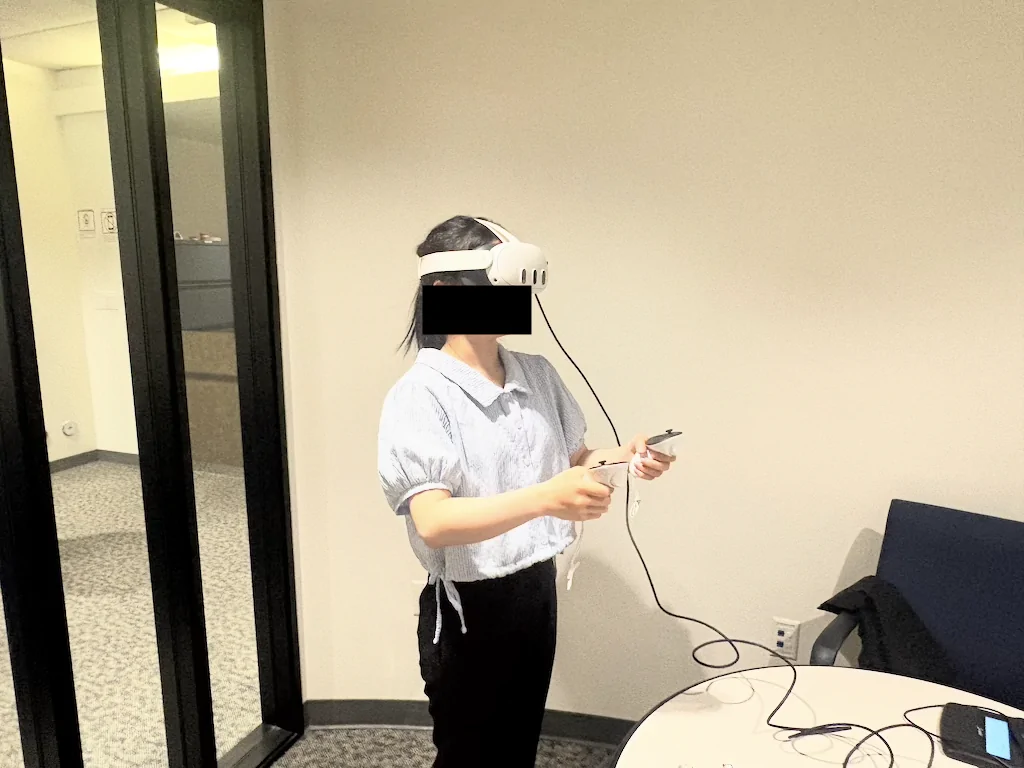
Demonstration of the immersive virtual reality system used for patient education in orthodontics in a cross-sectional feasibility and acceptability study at the university orthodontic clinic between August and September 2024. A volunteer, shown here, used a Meta Quest 3 headset and handheld controllers to navigate and interact with the virtual educational environment; this individual is not a study participant, and written consent to publish the image was obtained.

The immersive VR intervention is reported in accordance with the RATE-XR reporting guideline for the early-phase clinical evaluation of XR applications. Participants used a Meta Quest 3 headset and handheld controllers (Meta Platforms) to navigate and interact with the virtual environment (Figure 1). Head tracking allowed users to change their viewpoint naturally by moving their heads, whereas the handheld controllers were used to point, select, grab, and manipulate virtual objects. The VR educational experience was developed in Unity3D (Unity Technologies) and presented in a virtual consultation room environment. The application was developed in-house by the study team; each participant’s intraoral scan was exported from the intraoral scanner and imported into the Unity3D environment to generate a patient-specific 3D model.

**Figure 2.**
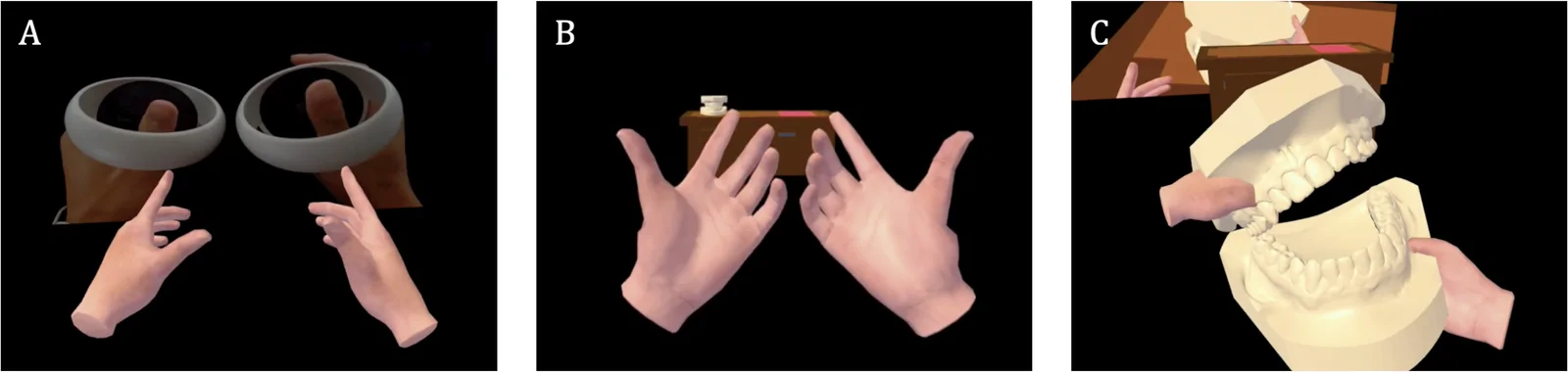
Screenshots of the immersive virtual reality educational workflow used in a cross-sectional feasibility and acceptability study of patient education in orthodontics (Meta Quest 3 and Unity3D). (A) Orientation and familiarization with the virtual environment and controller-based hand movements. (B) Navigation within the virtual consultation room toward the instructional desk. (C) Manipulation of the virtual dental models for 3D viewing and assessment of occlusion.

The experience incorporated an embedded orientation sequence in which participants first familiarized themselves with the virtual environment and controls before proceeding to educational content based on a 3D rendering of their own intraoral scan, representing their individual malocclusion (Figure 2A). This familiarization sequence included looking around the virtual environment, learning basic hand and controller movements, interacting with virtual objects, and moving within the virtual space. Participants were then guided to a virtual desk, where a 3D model of their dentition appeared (Figure 2B). They were prompted to manipulate the model, including holding it, rotating it, and viewing it from different angles; repositioning it; and positioning it in and out of occlusion (Figure 2C). The experience incorporated voice narration, stepwise prompts, and immediate audiovisual feedback to guide participant interaction. The sequence was fixed and identical for all participants, with no alternative modules or system versions.

Each participant completed the experience in a single session of approximately 15 minutes, which was administered and supervised one-on-one by a trained study investigator. Participants stood throughout, and the experience required only minimal whole-body movement, consisting mainly of head turning and hand-and-controller gestures within a cleared area of approximately 1.5×1.5 m. To prevent falls or collisions, a headset guardian boundary was configured, the surrounding area was cleared of obstacles, and the investigator remained beside the participant throughout to monitor for discomfort or cybersickness and to provide steadying support or assistance if needed. Participants were informed that they could pause or stop at any time; no formal stopping threshold was prespecified beyond the investigator’s judgment. Between participants, the headset and controllers were cleaned and disinfected as noncritical devices in accordance with standard dental infection-control protocols (consistent with American Dental Association and Centers for Disease Control and Prevention guidance), using an Environmental Protection Agency–registered hospital-grade disinfectant wipe. Setup required approximately 5 minutes per participant. Sessions were conducted in the clinic’s standard consultation room and delivered during the participant’s routine orthodontic visit rather than as a separate appointment. The VR application is an investigator-developed research prototype, is not publicly available, and has not been reviewed or cleared as a medical device by any regulatory body. It was used solely for research purposes.

### Measures and Covariates

After completing the VR activity, participants completed an investigator-developed postexperience questionnaire. The questionnaire included (1) closed-ended items on prior VR experience, interest in using VR for patient education in orthodontics, perceived educational utility, comparison with conventional viewing of orthodontic records, current preference, and preference under an improved technology scenario, scored on Likert-type response options (eg, “very interested/interested/neutral/uninterested/very uninterested” and “strongly agree/agree/neutral/disagree/strongly disagree”); (2) two numeric items rated on a 1-to-10 scale for overall experience with the VR equipment and the test room and ease of using the VR system; and (3) open-ended items soliciting positive and negative aspects of the experience. Beyond age and sex, no additional demographic or clinical covariates were collected. The preference items (items 6, 9, and 10) asked participants whether they preferred the VR experience or conventional 2D records viewed on a computer screen, reflecting the on-screen record viewing used in routine orthodontic care in our university orthodontic clinic. No physical dental models were used in this study. The questionnaire was developed by the investigators for the purpose of this feasibility study and was not formally validated. No validated usability instrument (eg, System Usability Scale), presence questionnaire, standardized cybersickness questionnaire (eg, Simulator Sickness Questionnaire), or objective knowledge-gain assessment were administered, and no pre-experience baseline knowledge assessment was conducted.

### Data Collection

Survey responses were collected through a web-based form completed on a study tablet in the clinic immediately after the VR experience. The form did not capture direct identifiers, and the analytic dataset contained no names or record numbers. However, because sessions were administered individually by a member of the study team, the investigator recorded each participant’s age and sex alongside their responses in the export. Responses were therefore deidentified rather than anonymous, and age and sex could be associated with individual responses. The supervising investigator was present in the clinic area while participants completed the survey; this is acknowledged as a potential source of social-desirability bias in the *Limitations* section.

### Conditions and Design

This was a single-group observational study. All participants received the same VR experience. There were no separate conditions or comparison groups, no randomization, and no masking. Because the study was not experimental, JARS (Journal Article Reporting Standards) reporting items that apply only to experiments are not applicable here, and reporting follows the JARS standards for nonexperimental designs.

### Data Diagnostics

All survey items were required in the web-based form, so there were no missing data and no postcollection exclusion of participants. No statistical outliers were removed, no data transformations were applied, and no imputation was required. Because the analysis was descriptive, no distributional modeling assumptions were required.

### Analytic Strategy

Nondata administrative summary rows generated by the survey platform were removed before analysis and no participant records were excluded. Closed-ended responses were summarized as counts and percentages with 95% Wilson score CIs. The 2 numeric items were summarized using means, SDs, medians, ranges, and 95% CIs based on the *t* distribution. Open-ended comments were reviewed and grouped into recurring descriptive themes by 2 investigators (YJK and GJ) by consensus; given the descriptive nature of the study, formal interrater reliability was not calculated. Because of the feasibility design and modest sample size, no inferential testing or formal subgroup analysis was performed. Analyses were conducted using descriptive statistical methods, and an exploratory descriptive cross-tabulation of reported interest against final preference was reported. No changes were made to the planned procedures, eligibility criteria, or analysis after the study commenced.

### Ethical Considerations

This study was reviewed and approved by the USC Institutional Review Board under protocol APP-24‐03274, which included the participation of minors. As a single-session, single-arm feasibility and acceptability survey rather than a clinical trial, the study was not prospectively registered. Adult participants (n=23) provided written informed consent prior to participation. Minor participants (n=5) provided written assent, and for each minor participant, a parent or legal guardian provided written permission for that specific minor’s participation, in accordance with the approved protocol. Participation was voluntary and had no effect on routine clinical care. No participant compensation was provided. Survey responses were deidentified at the point of export, and no identifiable participant information is presented in the analytic results. This paper’s figures and materials do not include identifiable images of participants.

## Results

### Participant Characteristics and Prior VR Experience

**Figure 3.**
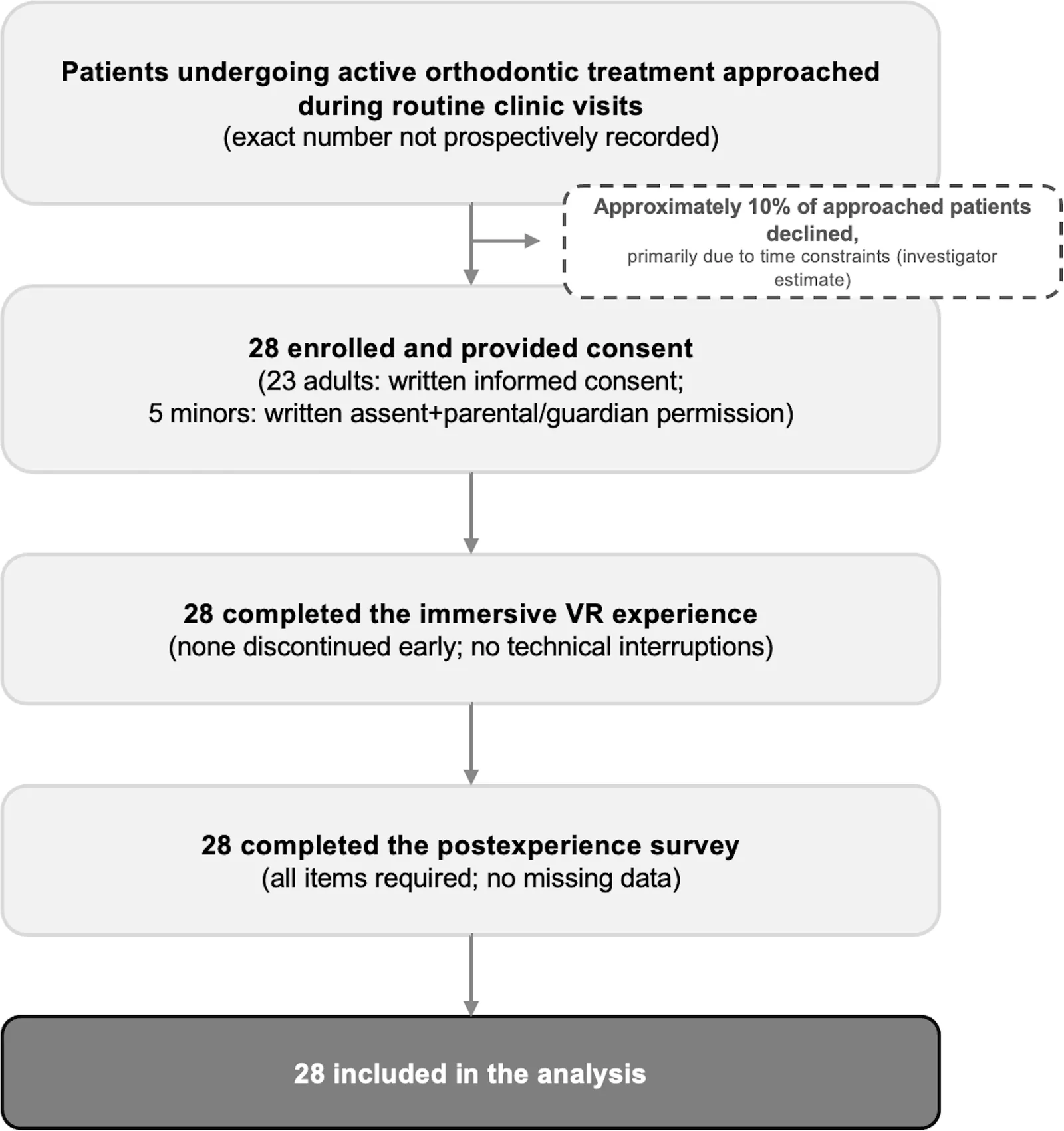
Participant flow through the cross-sectional feasibility and acceptability study at the university orthodontic clinic between August and September 2024. VR: virtual reality.

Among 28 respondents, 16 (57%) reported that they had never used VR before, 7 (25%) had some prior experience, and 5 (18%) described themselves as very comfortable with VR. Despite limited prior exposure among many participants, 22 (79%) respondents reported being interested or very interested in using VR to learn about their teeth and how their teeth fit and work together. Four (14%) respondents were neutral, and 2 (7%) were uninterested or very uninterested. Closed-ended survey responses are summarized in Table 1 and participant flow through the cross-sectional feasibility and acceptability study at the university orthodontic clinic is displayed in Figure 3.

**Table 1.** Distribution of closed-ended survey responses among patients undergoing orthodontic treatment (N=28) in a cross-sectional feasibility and acceptability study of an immersive virtual reality (VR) consultation room at the university orthodontic clinic between August and September 2024.^a^

Item and response	Patients, n (%)	95% CI (Wilson score)
Prior VR experience		
Never used VR before	16 (57)	39‐73
Some prior experience	7 (25)	12‐44
Very comfortable with VR	5 (18)	8‐36
Interest in using VR for learning		
Interested/very interested	22 (79)	60‐90
Neutral	4 (14)	6‐32
Uninterested/very uninterested	2 (7)	2‐23
VR can help patients learn about teeth/braces		
Agree/strongly agree	24 (86)	69‐94
Neutral	4 (14)	6‐32
VR better than 2D image records		
Yes	20 (71)	53‐85
No	8 (29)	15‐47
Current preference		
VR	19 (68)	49‐82
2D image records	9 (32)	18‐51
Future preference if technology improves		
VR	25 (89)	73‐96
2D image records	3 (11)	4‐27

a Percentages may not sum to 100 because of rounding.

### Perceived Educational Value and Preference

Out of 28 respondents, 24 (86%) agreed or strongly agreed that VR could help patients like themselves learn more about their teeth and what braces can do for them, whereas 4 (14%) respondents were neutral. When directly comparing VR with conventional 2D image records, 20 (71%) respondents felt that VR was a better way to learn, whereas 8 (29%) preferred conventional 2D image records.

With the current setup, 19 (68%) respondents preferred VR and 9 (32%) preferred conventional 2D image records. When asked whether they would prefer VR or conventional 2D image records if the technology were improved, 25 (89%) respondents selected VR and 3 (11%) selected conventional 2D image records. Because this latter item asked participants to imagine an unspecified improved system that was not actually tested, it is interpreted as a hypothetical-preference indicator rather than as an evaluation of an improved system.

### Overall Experience and Ease of Use

The mean overall experience rating for the VR equipment and test room was 7.9 (SD 1.6) on a 10-point scale (median 8, IQR 4‐10). The mean ease-of-use rating was 6.6 (SD 2.6; median 7, IQR 2‐10), indicating generally favorable but somewhat more variable perceptions of usability than of overall experience. Numeric survey ratings are summarized in Table 2.

**Table 2.** Numeric survey ratings (1‐10 scale) among patients undergoing orthodontic treatment (N=28) in a cross-sectional feasibility and acceptability study of an immersive virtual reality (VR) consultation room at the university orthodontic clinic between August and September 2024^a^.

Item	Rating, mean (SD)	Rating, median (IQR)	Rating, 95% CI
Overall experience with VR equipment/test room	7.9 (1.6)	8 (4-10)	7.3‐8.5
Ease of using the VR system	6.6 (2.6)	7 (2-10)	5.7‐7.6

a For overall experience, 10 indicates the best experience; for ease of use, higher scores indicate greater ease.

### Exploratory Descriptive Findings

An exploratory descriptive cross-tabulation of reported interest against preference for the current setup was examined, and both were measured in the same postexperience questionnaire. Among the 22 participants who reported being interested or very interested in using VR, 17 ultimately preferred VR and 5 preferred conventional 2D image records. Among the 6 participants who were neutral, uninterested, or very uninterested, 2 ultimately preferred VR and 4 preferred conventional 2D image records. This pattern is presented descriptively only; because interest and preference were both measured after the session, we cannot say that interest came first or predicted preference. Exploratory descriptive age-group patterns are summarized descriptively, among patients younger than 25 years, 77% (17/22) preferred VR and 23% (5/22) preferred conventional 2D image records. Among the 6 patients aged 25 years and older, 4 preferred conventional 2D image records and 2 preferred VR. Motion-related discomfort was reported only in participants aged 25 years and older. Because of the small subgroup sizes, these findings are presented descriptively only and should not be interpreted as evidence of an age effect.

### Safety and Tolerability

Out of 28 participants, 2 (7%) reported mild, transient discomfort consistent with motion-related symptoms in their open-ended responses (described as feeling “a little dizzy” while the environment loaded, and “light motion sickness” when moving within the environment). Both episodes were self-limited; neither required intervention; and no participant discontinued the session early. No other adverse events or technical interruptions requiring session termination were reported.

### Open-Ended Feedback

Open-ended comments were generally favorable. Common positive themes included the ability to visualize teeth and occlusion in 3 dimensions, direct interaction with the models, a sense of realism, and the novelty or fun of the experience. Participants frequently described the experience as interactive, cool, or engaging. Negative comments most often involved usability and hardware limitations. Several respondents noted that the controllers and navigation were difficult initially, whereas others mentioned lag, glitches, or insufficient responsiveness. A smaller number of participants described dizziness, motion sickness, or discomfort during the experience. Some participants also indicated that 2D image records still felt more intuitive in the current version of the system.

## Discussion

### Principal Findings

In this single-arm feasibility and acceptability study, immersive VR was well received as a patient-education modality in a university orthodontic clinic. Most respondents were interested in using VR to learn about their teeth, and a majority expressed a self-reported preference for VR over conventional 2D image records, even though most participants had no prior VR experience and the sample included both adolescents and adults. At the same time, ease-of-use ratings were relatively lower and more variable than overall experience ratings, and a minority of participants continued to prefer conventional 2D image records. These findings suggest that lack of prior familiarity with immersive technology may not prevent users from perceiving educational value, although prior experience may still influence aspects of immersion and usability [6,18,19]. Participants were generally able to engage with the experience and recognize its potential for learning, consistent with broader digital health and serious-games literature, showing positive perceptions of immersive technologies among end users [6,7,20]. Taken together, and interpreted in light of wide CIs, these findings provide preliminary evidence that immersive XR-based visualization is acceptable to patients undergoing orthodontic treatment and merits further, more rigorous evaluation.

### Comparison With Prior Work

Patients consistently highlighted interactivity, 3D visualization, and enjoyment as strengths of the VR consultation room. These impressions may have been supported by light gamified design features, such as stepwise task progression and immediate audiovisual feedback, which have been associated with improved engagement in educational settings [20,21]. Together, these features may support orthodontic communication by allowing patients to manipulate and inspect 3D dental models, an approach associated with improved spatial understanding relative to 2D formats in educational contexts [22]. This interpretation is consistent with prior orthodontic and dental literature emphasizing the importance of communication, visualization, and patient involvement in shaping the treatment experience [1,2], as well as with the broader serious-games and XR-in-health literature describing immersion and interactivity as drivers of engagement [20].

Our findings extend existing orthodontic XR research from professional education to patient-facing communication. Although previous orthodontic applications have primarily involved student education [15,16], and a recent scoping review identified patient engagement as an understudied use of XR in orthodontics [14], the favorable responses observed here are broadly consistent with patient-facing dental VR studies involving cleft care education, toothbrushing instruction, and dental anxiety [11-13]. They also align with reports that immersive VR is acceptable across clinical populations, including users with little prior VR experience [6,7,23]. The present study differs from these applications by allowing patients undergoing orthodontic treatment to view and manipulate their own intraoral scan to understand their individual malocclusion. At the same time, the usability and motion-comfort barriers we observed echo those reported for immersive VR more generally [24,25]. Overall, the findings provide preliminary evidence that patient-specific immersive visualization is acceptable in an orthodontic setting, while not establishing that it improves knowledge more effectively than conventional 2D image records.

### Interpretation

The study identified usability as an important implementation barrier. Although the overall experience rating was favorable, ease-of-use ratings were lower and more variable, suggesting that participants generally liked the experience but did not always find the system easy to use. This interpretation was supported by open-ended comments, which frequently referenced navigation difficulty, glitches, and motion-related discomfort. These observations likely explain why a subset of participants still preferred conventional 2D image records in the present setup and why preference for VR was higher under a hypothetically improved technology scenario. Prior VR literature has similarly identified usability, latency, and cybersickness-related discomfort as important barriers to immersive VR uptake [24], and qualitative syntheses report that users often tolerate such discomfort when they find the experience valuable [25]. Motion-related discomfort was uncommon and mild in this sample, with no early discontinuations, which is reassuring for feasibility; however, both reports occurred in participants aged 25 years or older. Because this subgroup was very small and age was analyzed only descriptively, no age effect can be inferred, and these observations should not be equated with findings from older-adult VR populations. General motion-comfort strategies, such as seated experiences, attention to session duration, and comfort-oriented locomotion, may be worth considering in future iterations [26]. However, the present data do not support age-based recommendations. Future development should prioritize interface simplification, system responsiveness, latency reduction, and mitigation of motion discomfort and should incorporate validated usability and presence instruments, such as the System Usability Scale, and a standardized cybersickness measure, such as the Simulator Sickness Questionnaire.

The preference findings in particular should be read as an acceptability signal rather than evidence of superiority. Preference for VR may reflect its perceived value, the novelty of an unfamiliar technology for most participants, and the appeal of interactive 3D visualization, as much as the intervention itself; order and novelty effects of this kind are well documented in immersive VR and can inflate short-term preference [27,28]. In addition, because continuous narration, stepwise prompts, and audiovisual feedback were delivered alongside active model manipulation, the experience cannot determine whether narration supported learning or competed for attention, consistent with split-attention and redundancy effects described in multimedia-learning and cognitive-load research [29]. The design features underlying these cautions, and the comparators needed to address them, are considered in the *Limitations* section.

### Limitations

This study should be interpreted as a feasibility and acceptability investigation, and several limitations should be considered. First, the sample size was modest (N=28), and the study was conducted in a single university orthodontic clinic, so generalizability is limited. The single-arm feasibility design and modest sample size precluded inferential testing or formal hypothesis evaluation; a retrospective power analysis was not performed because such analyses are not informative for already-completed studies. Instead, the present data are intended to inform sample size and effect size assumptions for a future controlled trial. Second, recruitment used consecutive convenience sampling with participation contingent on patient availability and willingness, introducing potential self-selection bias. Formal records of the number of patients approached and those who declined participation were not prospectively recorded. Third, VR was always experienced after participants’ routine prior exposure to conventional 2D image records, creating a fixed order, and the experience was novel for most participants; both order and novelty effects can inflate short-term preference; therefore, a counterbalanced or crossover design would be needed in future work to compare preferences reliably. Fourth, the study did not include an interactive flat-screen 3D comparator, so it cannot determine whether immersion specifically, rather than 3D interactivity in general, drove the favorable ratings; a future arm using an interactive flat-screen 3D model, ideally using each patient’s own scan, would isolate the contribution of immersion.

Fifth, the questionnaire was investigator-developed and was not formally validated; standardized usability, presence, and cybersickness instruments were not administered. No objective knowledge-gain measures were administered; therefore, the findings support acceptability, perceived educational value, self-rated experience, self-rated ease of use, and self-reported preference only, and not learning effectiveness or superiority. Sixth, no baseline knowledge was assessed before VR exposure, so perceived educational value cannot distinguish new learning from confirmation of prior understanding. Seventh, the forced-choice preference items did not allow participants to indicate equal preference, no preference, or uncertainty, which may have overstated the apparent strength of preferences. Eighth, the postexperience survey was completed with the supervising investigator present, introducing potential social-desirability bias. Ninth, although age and sex were recorded, the sample size was too small to support robust inferential subgroup analyses, and age-related findings should be interpreted as exploratory and descriptive. Finally, the comparison with conventional 2D image records was a self-reported preference rather than a controlled comparison. Therefore, future studies should incorporate pretest-posttest designs, validated patient-reported outcome measures, validated XR usability and cybersickness instruments, and direct comparisons with established educational approaches and variations in narration load alongside interaction.

### Conclusions

Patients undergoing orthodontic treatment in this cross-sectional feasibility and acceptability study were generally receptive to an immersive VR consultation room that presented each patient’s own intraoral scan models for patient education in orthodontics. Most participants believed that VR could help patients better understand their teeth and orthodontic treatment, and many valued its interactivity, 3D visualization, and engaging format. This study is novel in evaluating patient-facing immersive VR specifically within orthodontics—an application that remains understudied relative to VR training for students and clinicians. However, usability issues, motion discomfort, and technical limitations remain important barriers to broader adoption. Because the design was single arm and the comparison with conventional 2D image records reflected self-reported preferences rather than a controlled comparison, the results should be regarded as preliminary and hypothesis-generating rather than evidence of educational effectiveness. If these acceptability signals are confirmed in more rigorous studies, immersive VR may have a practical role as an adjunct to orthodontic patient communication, supported by future controlled studies that incorporate validated usability, presence, and cybersickness instruments and objective measures of knowledge gain.

## Supplementary material

10.2196/102686 Multimedia Appendix 1 Postexperience survey instrument (Virtual Reality Test Room Survey).

## Abbreviations

JARS: Journal Article Reporting Standards
RATE-XR: Reporting for the early-phase clinical evaluation of applications using extended reality
USC: University of Southern California
VR: virtual reality
XR: extended reality
