# Supplementary material for: Immersive Virtual Reality for Patient Education in Orthodontics: Cross-Sectional Feasibility and Acceptability Study
Source: JMIR Serious Games. 2026 Aug 27;14:e102686. doi: 10.2196/102686 (PMC13521208; doi:10.2196/102686)
Supplement: Supplementary file 1 [file games-v14-e102686-s001.docx]

**Multimedia Appendix 1. Post-Experience Survey Instrument**

*Virtual Reality Test Room Survey*

This appendix reproduces the investigator-developed post-experience questionnaire administered to participants immediately after the immersive virtual reality (VR) educational experience. The instrument was created by the study investigators for the purposes of this feasibility and acceptability study and was not formally validated. It was administered electronically using a web-based form. All items were required. Items 1 through 3 used Likert-type response options, items 4 and 5 used 10-point numeric rating scales, items 6, 9, and 10 used forced-choice options, and items 7 and 8 were open-ended free-text responses.

**Survey Items**

**Item 1.** Prior to this virtual test room, did you have any experience with virtual reality (VR) technology?

*Response format:* Single choice

- Never used any form of VR before
- Some prior experience with VR
- Very comfortable with VR

**Item 2.** How would you feel about using VR when learning about your teeth and how they fit and work together?

*Response format:* Single choice (5-point Likert)

- Very uninterested
- Uninterested
- Neutral
- Interested
- Very interested

**Item 3.** Do you think VR can help patients like yourself learn more about their teeth and what braces can do for them?

*Response format:* Single choice (5-point Likert)

- Strongly disagree
- Disagree
- Neutral
- Agree
- Strongly agree

**Item 4.** On a scale of 1 to 10, with 10 being the best, how do you rate your experience with the VR equipment and the test room?

*Response format:* Numeric rating scale, 1 to 10 (10 = best)

**Item 5.** On a scale of 1 to 10, with 1 being the hardest, how easy or difficult was it to use the VR system?

*Response format:* Numeric rating scale, 1 to 10 (1 = hardest)

**Item 6.** Do you think using VR was a better way to learn about your teeth than 2D images on a screen?

*Response format:* Single choice

- Learning in VR is better
- Learning from computer screen is better

**Item 7.** What did you like about the VR experience?

*Response format:* Open-ended free text

**Item 8.** What did you not like about the VR experience?

*Response format:* Open-ended free text

**Item 9.** With how the set-up is currently, would you rather look at models on VR or on a screen?

*Response format:* Single choice

- VR
- Computer screen

**Item 10.** With improvements in VR technology, would you rather use VR to learn about your teeth or on a screen?

*Response format:* Single choice

- VR
- Computer screen

***Note:*** *Item wording and response options are reproduced as administered. The questionnaire was developed by the study investigators and was not formally validated; this limitation is described in the main manuscript.*
